# Effective Microorganism Solution and High Volume of Fly Ash Blended Sustainable Bio-Concrete

**DOI:** 10.3390/biomimetics7020065

**Published:** 2022-05-23

**Authors:** Ghasan Fahim Huseien, Ali Taha Saleh, Sib K. Ghoshal

**Affiliations:** 1 Department of the Built Environment, College of Design and Engineering, National University of Singapore, Singapore 117566, Singapore; 2 Department of Chemistry, College of Sciences, University of Misan, Amarah 62001, Iraq; alitaldosari@uomisan.edu.iq; 3 Department of Physics and Laser Centre, AOMRG, Faculty of Science, Universiti Teknologi Malaysia, Skudai 81310, Malaysia

**Keywords:** effective microorganism, bio-concrete, fly ash, acid resistance, sustainability

## Abstract

Currently, the production of sustainable concrete with high strength, durability, and fewer environmental problems has become a priority of concrete industries worldwide. Based on this fact, the effective microorganism (EM) solution was included in the concrete mixtures to modify the engineering properties. Concrete specimens prepared with 50% fly ash (FA) as an ordinary Portland cement (OPC) replacement were considered as the control sample. The influence of EM solution inclusion (at various contents of 0, 5, 10, 15, 20, and 25% weight) in the cement matrix as water replacement was examined to determine the optimum ratio that can enhance the early and late strength of the proposed bio-concrete. The compressive strength, porosity, carbonation depth, resistance to sulphuric acid attack, and the environmental benefits of the prepared bio-concrete were evaluated. The results showed that the mechanical properties and durability performance of the bio-concrete were improved due to the addition of EM and FA. Furthermore, the inclusion of 10% EM could increase the compressive strength of the bio-concrete at 3 (early) and 28 days by 42.5% and 14.6%, respectively. The durability performance revealed a similar trend wherein the addition of 50% FA and 10% EM into the bio-concrete could improve its resistance against acid attack by 35.1% compared to the control specimen. The concrete mix designed with 10% EM was discerned to be optimum, with approximately 49.3% lower carbon dioxide emission compared to traditional cement.

## 1. Introduction

In recent years, various types of effective microorganisms (EMs) have been used to produce some innovative bio-concretes for construction applications [1,2,3]. For the manufacturing of bio-concrete, several effective biological strategies and methods are adopted to implement these EMs into the cement matrix [4,5]. Generally, such EMs in water, soil, industrial wastewater effluent, oil reservoirs, and acidic hot springs are quite abundant. Thus, the EMs can potentially be used in the mix design to make high-performance, durable, and environmentally-friendly bio-concretes [6,7]. Construction industries worldwide have classified the solutions of EMs into three main types based on bacteria, viruses, and fungi. It was demonstrated that certain bacteria in the EM solution enable the precipitation of different elements or chemical compounds, which are useful for the production of eco-friendly and durable bio-concretes [8,9]. Diverse methods have been developed for the successful incorporation of EM solution into the fresh concrete mixes, such as the direct addition of microbial broth; the inclusion as spores, in the form of immobilized structures into the silica gels or activated carbon, in the form of micro- and nano-encapsulation, or using a chemical reaction-mediated vascular network with uniform/homogeneous distributions of the microorganisms [4,10,11].

Isa et al. [12] found that the inclusion of 3% EM solution weight into the cement can improve its compressive strength (CS) by 14.1% at 28 days of curing age. The observed enhancement in the CS was attributed to the higher hydration that filled the concrete pores with C-(A)-S-H gels [13]. It was reported [14] that bacteria devoid of food/nutrients in the EM solutions are unable to improve the CS performance of the concrete when included. Sato et al. [15] showed that the use of EM solution could improve the CS compared to the traditional cement-based control specimen. The inclusion of 5% EM in the concrete matrix was shown to be optimal for achieving its elevated CS than the one devoid of EM solution [16]. The CS of the concrete (43.2 MPa) prepared with 5% EM was about 42.9% greater than the specimen devoid of EM solution. The concrete made with 5% EM attained 54% of its ultimate strength in 1 day, indicating the usefulness of EM solution in enhancing the early strength desirably. The splitting tensile strength (STS) and flexural strength (FS) of the concrete were also improved due to the addition of EM solution into the mixes. The EM solution (5, 10, and 15% as water replacement) self-compacting concretes were designed by Rizwan et al. [17] to determine their effects on the enhanced strength properties, plastic viscosity, setting times, hydration kinetics, and volume stability together with decreased water absorption of the proposed cement pastes in a hardened state. Many studies [18,19,20,21,22] acknowledged that the addition of EM solution into the concrete matrix could improve its resistance to corrosion attacks. Briefly, for structural purposes, the modified bio-concretes containing EM solutions generated renewed interest in the construction industries because of their excellent workability, mechanical, and durable performances. 

Looking at the immense advantages of the EM solution-incorporated modified bio-concretes, we proposed some eco-friendly high-performance concrete mixes containing EM solution and FA that can withstand aggressive environments, including sulphuric acid attacks. The optimum content of EM solution that can improve the early and late strength performance of the FA-cement-modified bio-concrete was determined. The CS values of the modified bio-concrete mixes were measured at various curing ages (3, 7, 28, and 56 days). The benefits of FA/EM solution incorporation into the bio-concrete for enhancing their durability properties in terms of the resistance against sulphuric acid attack, porosity, and carbonation depth were analyzed. In addition, the environmental benefits of the proposed eco-friendly sustainable bio-concretes containing FA/EM were evaluated to demonstrate the reduction in CO_2_ emissions.

## 2. Materials and Methods

### 2.1. Materials and Mix Design

In this study, the EM solution (as a tap water replacement) activated bio-concretes were prepared using high volume FA as OPC replacement. FA class F (low calcium content) was obtained from a local power plant (Johor, Malaysia) and used without further chemical or physical treatment. The chemical composition of FA (Table 1) was analyzed by X-ray fluorescence spectroscopy (XRF) and was composed of silica oxide (SiO_2_) and aluminum oxide (Al_2_O_3_), contributing more than 86% of the total weight. OPC was obtained from a local supplier and used as the main resource for calcium oxide (CaO), which was mainly composed of CaO and SiO_2_ (82%). In the bio-concrete mixes, 50% of OPC was replaced by FA to reduce the CaO and increase the Al_2_O_3_ contents, thus improving the ratio of SiO_2_ to Al_2_O_3_ and CaO to Al_2_O_3_.

Table 2 shows the physical characteristics of OPC and FA, wherein FA displayed lower specific gravity (2.2) compared to OPC (3.15), and the appearance of FA and OPC was grey and dark grey, respectively. The percentage passing through a 45 μm (No. 325) wet sieve for OPC and FA was, correspondingly, 90% and 100%, in accordance with ASTM C430.

**Figure 1:**
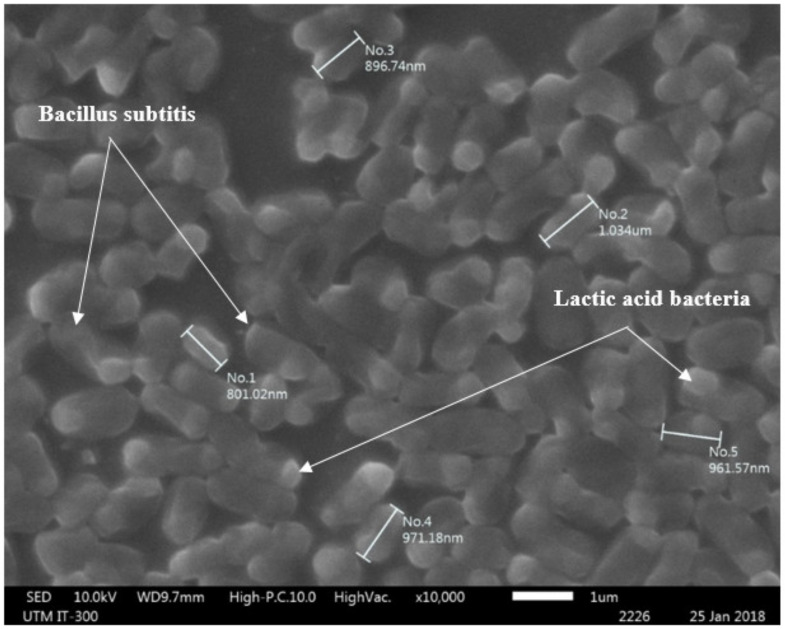
SEM image of the Bacillus subtilis and lactic acid bacteria in EM solutions.

In this study, 5 kg of type 1 EM (EM-1) was procured in a closed vessel (Johor, Malaysia), and 5% of it was activated using 90% water and 5% blackstrap molasses as the primary food source. As shown in Figure 1, the EM solution was comprised of *Bacillus subtilis* and lactic acid bacteria. The activated solution was made (with a pH of less than 4) using fermentation for 7–10 days without an oxygen supply. The existence of the EM in the solution and its solubility in water were examined before implementation into the concrete mixes. The color appearance of the produced bio-concrete (fresh and hardened phases) was analyzed. Initially, EM showed a dark brown hue, then transformed to dark yellowish after substituting with 10% of water, and finally became homogeneous light brown after substituting with 25% of water. Similar findings regarding the EM solution colors were reported elsewhere [23].

Table 3 presents the compositions of the modified concrete. Later, these ratios were altered to achieve the desired CS of 30 MPa at the curing age of 28 days. Next, 50% of OPC was replaced by FA to determine the optimum EM solution content in enhancing the early and late strength performance of the bio-concrete. For this purpose, 5, 10, 15, 20, and 25% of EM were used to replace the tap water by weight percentage. The water to cement ratio (w/c) was fixed at 0.55 for all mix designs. The OPC-FA-EM mixes were cast at various ratios of the constituents wherein the fine and coarse aggregates were mixed first for 3 min to achieve a homogenous mixture, and then the binders (OPC and FA) were added followed by mixing for an extra 3 min. Afterward, the solution (water or water/EM) was included and mixed for 2 min. Then, the fresh concrete was poured into cubical and cylindrical molds in two layers and subjected to a vibration table for 15 s to reduce the air voids. The produced concrete specimens were cured for 7 days before assessing their strength performance, thus determining the optimum EM content suitable for the batch design of the proposed bio-concrete.

### 2.2. Test Procedure

Several tests were adopted to evaluate the sustainability performance of the suggested bio-concrete, including the CS, porosity, carbonation depth (CD), resistance against sulphuric acid attack, and carbon dioxide emission. For the CS evaluation, cubic specimens with size (100 mm × 100 mm × 100 mm) were made and recorded at various curing ages (3, 7, 28, and 56 days). For each age, three specimens were tested to find the statistical average. The CS test was conducted following the ASTM C109 and ASTM C150 stipulations. Following the ASTM C642 standard, the porosity and total voids of the disc-shaped specimens (100 mm diameter and 50 mm depth) were obtained. The BS 1881 standard was followed to measure the CD of the cylindrical specimens (100 mm diameter and 200 mm depth). Regarding the durability performance in an aggressive environment, modified bio-concretes were immersed in H_2_SO_4_ solution (10% concentration), and the pH of the acid solution was maintained for 2 months. The immersed specimens were tested after 180 and 365 days to measure their strength loss, weight loss, internal cracks, ultrasonic pulse velocity (UPV) readings, visual appearance, and surface morphologies. 

The environmental benefits of the FA and EM-incorporated bio-concretes were evaluated in terms of CO_2_ emission levels. For this assessment, each material (OPC, FA, and EM) was considered over the entire life cycle, including the production, transport cost, and other chemical treatments in the laboratory. The distance of transportation for FA and OPC was 35 km and 5 km, respectively. Regarding the life cycle of each constituent of the bio-concrete, the net CO_2_ release was calculated depending on the production, transport distance, and fuel consumption to procure each component. For each ton of OPC, FA, and EM, the corresponding net CO_2_ release was 0.904, 0.012, and 0.00001 ton/ton. For every cubic meter of the constituent material, the net CO_2_ emission was calculated via:

(1) $$YG=\overset{n}{\underset{i=1}{\sum }}xi\;\left[\left(di\times fi\times zi\right)\right]$$

where *Y_G_* is the net CO_2_ release (ton/ton), *xi* is the mass of constituent *i* (ton/m^3^*)*, *di* is the transportation distance (km), *fi* is the diesel usage (liter/km), *zi* is the CO_2_ release (ton) for 1 L diesel burning.

## 3. Results and Discussion 

### 3.1. Microstructure Properties of FA and OPC

**Figure 2:**
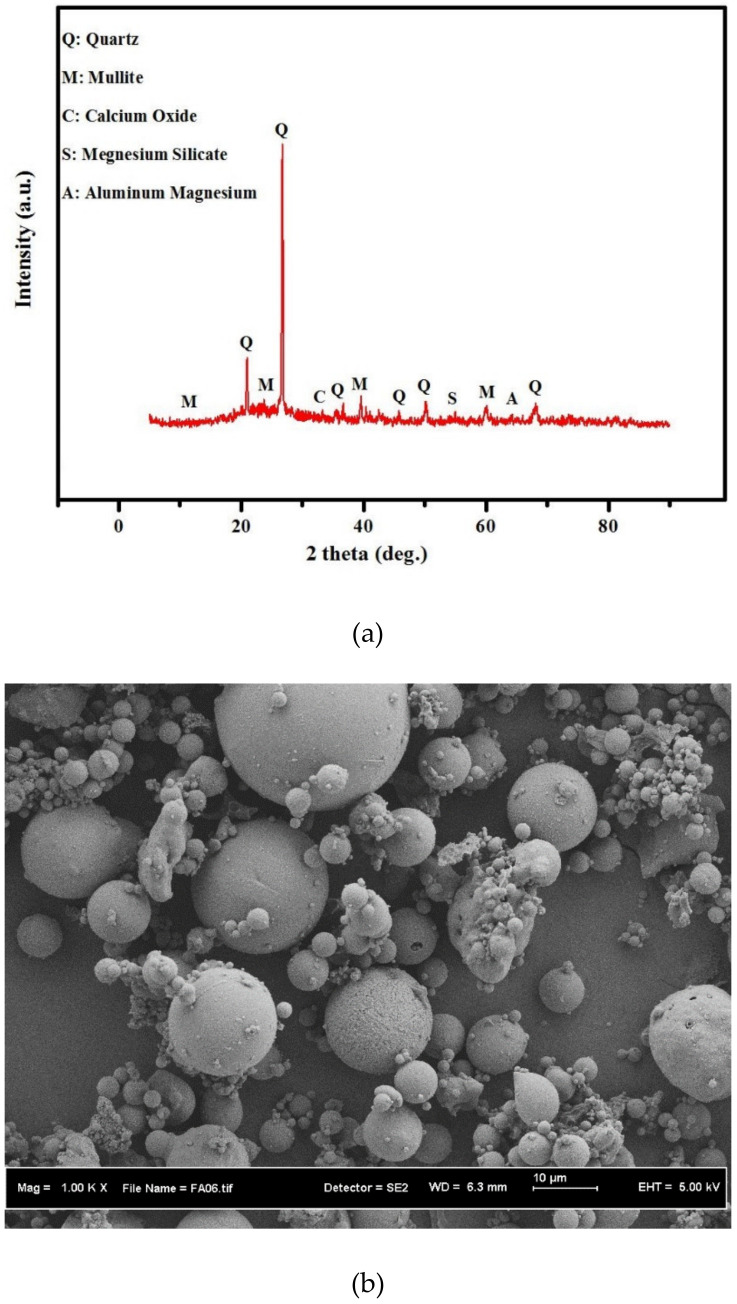
Mineral properties of FA powder (**a**) XRD profile and (**b**) SEM micrograph.

Figure 2 displays the XRD profile and SEM image of FA. The XRD profile of FA (Figure 2a) was comprised of many sharp peaks corresponding to the mullite, quartz, silica, and alumina crystalline structures. SEM image of FA (Figure 2b) revealed the existence of spherical blobs (medium size of 10 µm smaller than OPC particles) with smooth surface morphology. 

**Figure 3:**
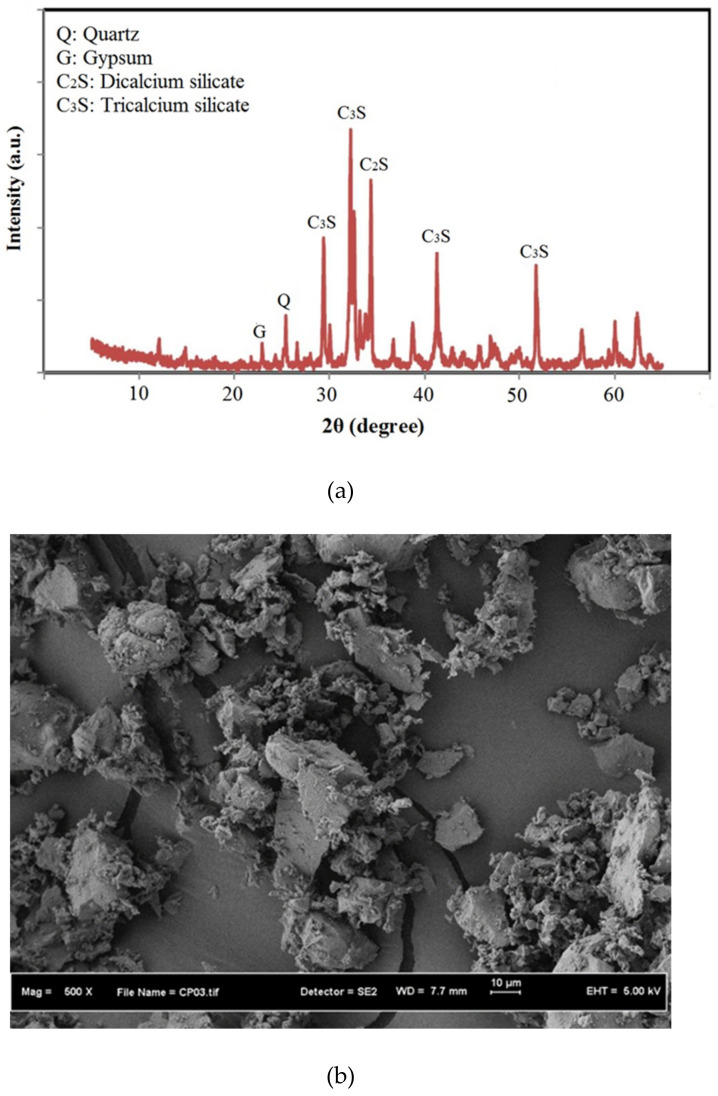
Mineral properties of OPC powder (**a**) XRD pattern and (**b**) SEM image.

Figure 3 illustrates the XRD profiles and SEM micrograph of OPC. The broad hunch in the region (25° to 55°) indicated the amorphous phase of OPC (Figure 3a). In addition, the XRD pattern of OPC exhibited sharp crystalline peaks of Dicalcium Silicate (C_2_S) and Tricalcium Silicate (C_3_S). The non-crystalline silicate minerals in FA reacted with Ca(OH)_2_ to create the cement hydration products such as calcium silicate hydrate (C-S-H) gels. The reduction in Ca(OH)_2_ and increase in C-S-H gel led to the enhanced strength performance and durability of the bio-concrete. The SEM image analyses (Figure 3b) of OPC showed the presence of irregularly shaped particles with a median particle size of 14.6 µm, which was larger than the FA particles. This indicated that the reactivity of FA as a pozzolanic material was higher compared to OPC due to their larger surface area with tinier particle size.

### 3.2. Surface Tension, pH and Viscosity of EM Solution

The effect of EM solution replacement for water on the pH values of the produced bio-concretes was measured (Table 4). The substitution of the tap water by 5 to 20% of the EM solution caused a reduction in the water pH from 6.7 to 4.0, respectively. The pH of EM (3.5) significantly affected the pH of the prepared solution (water-EM). Isa et al. [12] and Andrew et al. [16] observed a similar trend in the prepared solution PH variation where the influence of EM on the pH was significant. The fresh EM solution pH was reported to be more than 3.5 [24]. Cabrera et al. [25] reported the significant effects of high and low pH water on the fresh and hardened properties of concretes. In this work, the results revealed that pH level played a critical role in the process of hydration, early and late strength, as well as durability performance of the concrete. The EM content (Table 4) also appreciably affected the viscosity of the water-EM solution, which was increased from 0.95 to 1.25 mPa with the increase in water substitution by EM from 0 to 25%, respectively. The highly viscous nature of EM (1.44 mPa) could significantly affect the viscosity of the prepared solution. In a study according to Wang and Li [26], the mixing water viscosity can affect the fresh and hardened characteristics of the concretes. This can help to produce fluid concrete with high cohesiveness to decrease the bleeding, segregations, and settlements. In addition, the workability and durability performance of the concretes can be improved by regulating the pH of the solution [27]. In the current study, the observed increase in the EM solution viscosity could be ascribed to the inclusion of molasses during the mixture preparation.

The fresh and hardened characteristics of the concrete were significantly influenced by the surface tension of the mixed solution (Table 4), wherein the prepared solution surface tension values were decreased with the increase in EM levels during water substitution. With the increase in EM content from 0 to 25%, the corresponding surface tension values dropped from 66 to 39.9 mN/m. This was mainly due to the considerable influence of the low surface tension of EM (35.6 mN/m) on the surface tension of the prepared solution. The liquid surface tension describes the attraction at the interfacial region between two phases, such as liquid–solid or liquid–vapor or liquid–gas or liquid–air. The surface tension characterizes the elastic trend of any fluid surface that allows it to achieve the lowest possible surface area. This plays a crucial role in the capillary actions occurring during the release of fluid that makes contact with the porous materials (rock or mineral). The interfacial tension is roughly similar to the surface tension in terms of cohesive force involvement. In this work, the surface tension of the solution was important to achieve the superior characteristics of the proposed bio-concretes.

### 3.3. Compressive Strength

**Figure 4:**
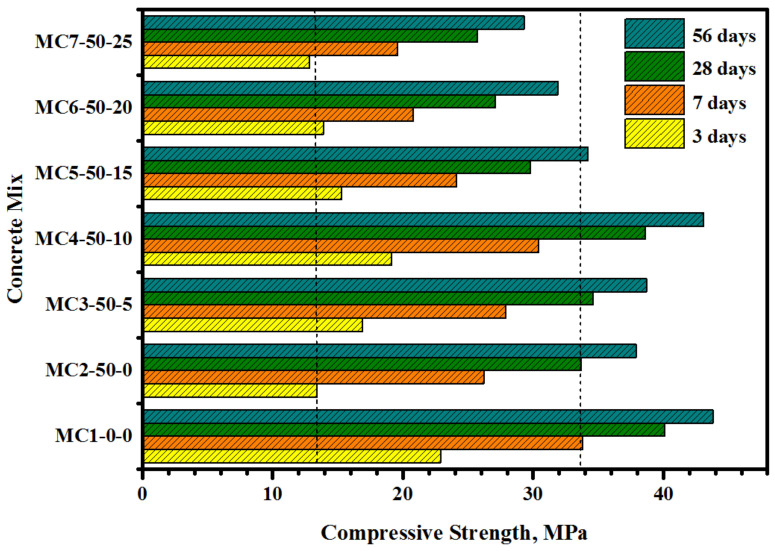
Influence of EM solution on the CS development of bio-concrete.

Figure 4 displays the effect of FA/EM on the CS performance of the concrete at early and late ages, wherein the addition of a high volume of FA (50%) caused significant effects on the development of the CS. At an early age (3 days), the replacement of cement by 50% of FA led to a drop in the CS value from 22.9 to 13.4 MPa with a loss in strength above 41%. However, the loss of strength was decreased to 22.4 to 13.4% by increasing the curing age from 7 to 56 days, respectively. Several studies [28,29,30,31,32] showed that the replacement of cement by FA could increase the SiO_2_ and Al_2_O_3_ levels with a reduction in CaO content, thus affecting the cement hydration process. The incorporation of FA into the cement matrix caused significant effects on ion transport and gel formation, fluctuating the electrochemical properties, as observed from the impedance analyses [33,34]. Meanwhile, cement substitution with a high volume of FA reduced the hydration process, thus affecting the net C-S-H gel formation and making the bio-concretes more porous with reduced CS performance. The reduction in the strength loss percentage of the bio-concretes by increasing the curing ages from 3 to 56 days can be attributed to the improvement of the hydration of alite (Ca_3_SiO_5_), belite (Ca_2_SiO_4_), and tricalcium aluminate (Ca_3_Al_2_O_4_) [35,36]. The observed enhancement in the CS values of the cement-FA specimens after 28 days was mainly due to the formation of extra C-S-H gels originating from alite and belite [37], together with the secondary to extra FA-mediated C-S-H gel formation [38,39]. Conversely, the addition of the high volume FA in the bio-concrete could enhance the CS value after 28 days, mainly due to various hydration products resulting from the hydraulic reactions with Ca(OH)_2_ that existed in the cement. However, the increased curing time enhanced the CS value of the specimens; this increase was mainly due to the pozzolanic reactions that enabled the C-S-H gel formation in contrast to the control specimen at 7 days [40,41,42].

The effect of the EM solution on the early and late CS development of the cement-FA concretes was evaluated (Figure 3). The inclusion of the EM solution into the cement-FA matrix significantly affected the strength and the CS values, which were increased with increased curing age. At 3 days of age (early), the CS values of the bio-concrete were increased from 13.4 to 19.1 MPa, with the corresponding increase in the EM solution content from 0 to 10% as a water replacement. However, with the further increase in EM solution from 15 to 25%, the corresponding CS values dropped from 15.3 to 12.8 MPa. At 28 days (late age), the CS values were increased from 33.7 to 38.6 MPa, with the increase in the EM solution content from 0 to 10%, respectively. Furthermore, the CS values were reduced from 29.8 to 25.7 MPa, with an increase in the EM solution from 15 to 25%, respectively. Irrespective of the curing ages (3 to 56 days), the concrete produced with 10% of EM solution displayed the optimum CS development. These results agreed with the other findings on early age CS development, which was mainly due to the reduction in the hydration process and ineffective filling of pores by the molasses. The pH value of the original concrete was below the control specimen, indicating the lower alkalinity of the concrete at early ages. It was acknowledged that the hydration reaction could be negatively affected by the reduction in both pores and fluid alkalinity, yielding a reduced CS of the concrete at early ages [41]. In addition, the cement hydration process can be delayed because a low pH can obstruct microstructure development in the cement pastes at early ages [43].

The early-age CS values of the modified concrete were directly influenced by the pH of the solution. It was shown [44] that the absorption of water molecules in the interfacial region comprising both water and solid could generate organic molecular films that, in turn, lowered the interfacial energy of the cement particles and hydration products, hindering the progress of the hydration reactions and thereby affecting the CS development of the concretes. As a result, both the alkali content and solution pH were lowered, thus delaying the hydration process of the alite in the concrete. Another factor that caused the reduction in the CS development at an early age was related to inadequate pore filling by the molasses that decelerated the hydration reaction in the cement matrix. This delay in the hydration reaction mechanism may be due to the molasses adsorption on the hydrated cement particles and/or hydration product surfaces [45]. Additionally, the delay in the hydration process can be ascribed to the increase in ion solubility aroused from the cement particles and their successive adsorption by Ca(OH)_2_ and hydrated C–S–H gels, restricting their expansion in the concrete network. Similar results were obtained by Ali et al. [46], who showed an increase in the CS values at all ages (except at early ages) of the molasses-incorporated concretes.

The acceleration of the hydration process enabled efficient pore filling by C-(A)-S-H gels, thus achieving a better strength performance in the bio-concrete made with 5 and 10% EM than the control specimen. Moreover, the low value of the surface tension of the mixed water was responsible for the better performance of the bio-concrete, wherein very tiny pores were formed by the water when mixed in the concrete. It was inferred that the interaction amid the capillary walls could be controlled by lowering the solution surface tension because the capillary pores’ diameter is reduced to micro-scale during drying [47]. In addition, the surface energy can be lowered by controlling the mixed water surface tension adsorbed on the cement particles’ surface [48]. Thus, harmless voids/pores are enhanced, and that leads to the creation of high-density network structures. The CS values of the proposed bio-concretes were improved by 25–30% with the decrease in the mixed water surface tension than the standard concrete [49]. A study showed that [50] the performance of the admixtures can be improved by lowering the surface tension of the water rather than adding excess mixing water. 

The inclusion of EM at high amounts could reduce the solution surface tension, thus reducing the CS values of the bio-concretes [48]. The flowability of the bio-concrete prepared with 20% EM was reduced due to the inhomogeneity of the mixes and little bleeding, achieving very poor hydration. Additionally, the particle separation generated a small number of chemical bonds within the concrete matrix, producing lower pH and CS values for the concrete containing 20% EM. Other factors responsible for the lower CS values may be related to the high volume of molasses. Akar and Canbaz [51] showed that lower CS at higher EM levels might be due to the late production of Ca_3_SiO_4_ from the hydration reaction, which is vital for the CS development of the bio-concrete. However, at higher molasses contents, the ettringite formation was accelerated, averting the second hydration of the aluminates phase and delaying the hydration of the Ca_3_SiO_4_ [44].

### 3.4. Microstructure Properties

**Figure 5:**
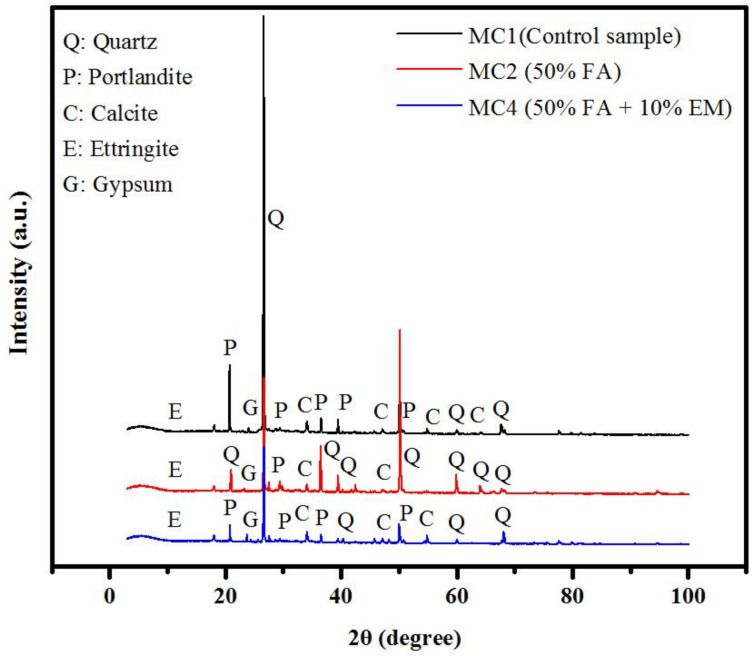
Effect of FA and EM content on XRD of prepared concrete specimens.

The resulting analysis of the XRD at 28 days of curing age for the modified concrete with FA and EM compared to the control samples is presented in Figure 5. The results show that the inclusion of a high volume of FA (50%) as a cement replacement negatively affects the hydration process and restricts the total formulation of Ca(OH)_2_, CaCO_3_, and C-(A)-S-H gels. The results show that the increasing content of FA resulted in more non-reacted silica, as displayed by the peaks at 20.1, 39.8, 50, and 60.4°. These consisted of sharp crystalline peaks corresponding to the quartz (SiO_2_), portlandite (CaOH)_2_, calcite (CaCO_3_), ettringite (Ca_6_Al_2_(SO_4_)3(OH)_12_·26H_2_O), and gypsum (CaSiO_4_) phases that resulted from the binary blends (OPC and FA). It was found the intensity of the quartz peaks trend to increase with the increasing FA level from 0 to 50% as an OPC replacement, wherein more quartz became nonreactive at 50% of FA (MC2-50-0) compared to the control sample (MC1-0-0), which resulted in lower strength. These products were generated from the reaction between the amorphous SiO_2_ and Al_2_O_3_ from FA with highly crystalline C_3_S, C_2_S, and C_3_A from OPC phases. Conversely, the concrete made without OPC showed portlandite and calcite peaks around 18-65°. However, by replacing OPC with 50% FA, the portlandite peaks in the range of 18–50° became lower. Unlike with the inclusion of 10% of EM in the OPC-FA matrix modified concrete specimens (MC4-50-10), the quartz peak intensity was decreased at the expense of the portlandite peak intensity as more silica dissolved and created the C-(A)-S-H gels in the hydration process (Table 5). Briefly, the XRD results clearly revealed the influence of the EM and FA on the generated C-S-H gel and the strength of the prepared modified concretes. 

**Figure 6:**
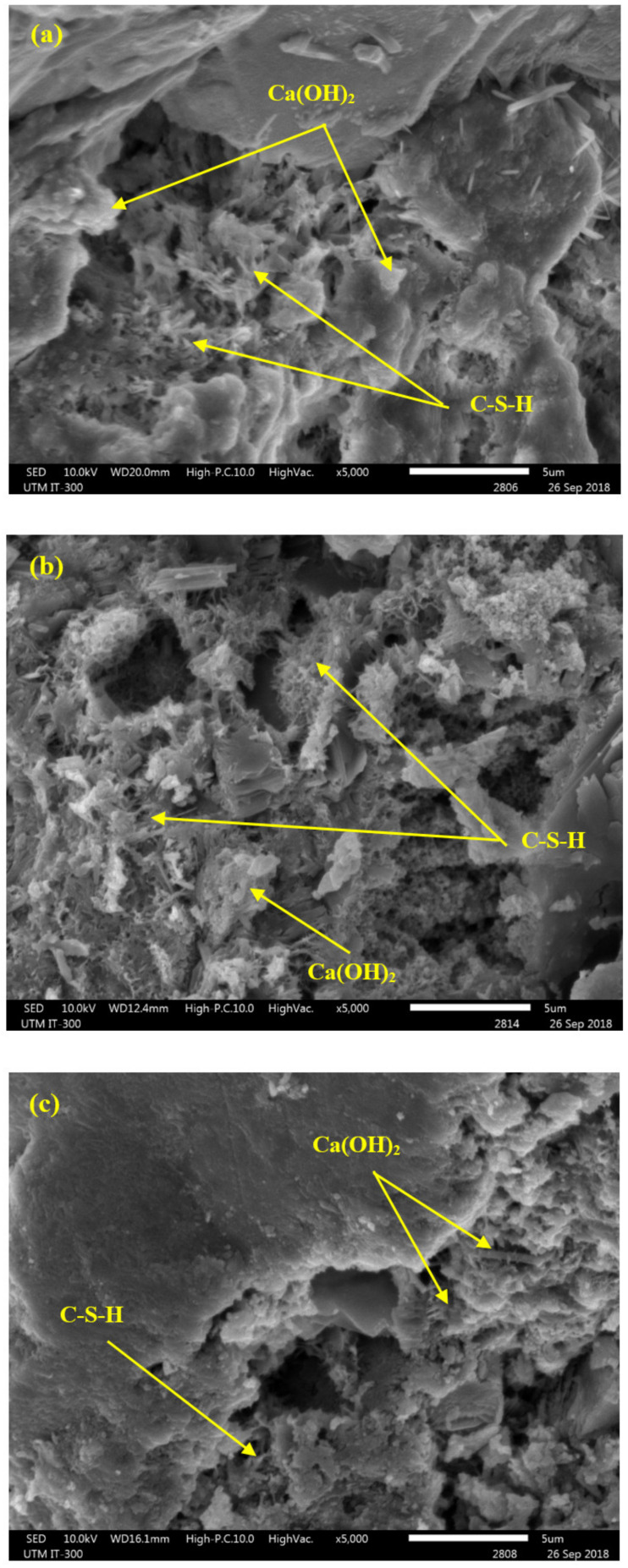
SEM images of (**a**) MC1-0-0, (**b**) MC2-50-0 and (**c**) MC4-50-10 at 28 days.

Figure 6 shows the SEM images of MC1-0-0, MC2-50-0, and MC4-50-10 specimens at 28 days. The alteration in the surface morphology of the bio-concrete clearly showed the impact of the FA and EM inclusion in the cement matrix. Compared to the control specimens (Figure 6a), the FA and EM-based concretes (Figure 6b,c) led to improved hydration processes and produced extra C-(A)-S-H gels, thus enhancing the CS values of the concretes. Generally, the concrete containing FA (Figure 6b) showed less dense microstructures than the cement-FA-EM concrete, which was due to the lack of C–S–H gel formulation. The bio-concrete designed with 10% EM and 50% FA showed dense microstructures with enhanced morphology (Figure 6c). In addition, the morphologies of the bio-concretes revealed the presence of a small number of unreacted particles. The enhanced EM solution-mediated hydration reaction and aluminum content were mainly responsible for the improvement to the CS of the bio-concrete, which led to the formulation of more (C-(A)-S-H) gels compared to the specimen designed only with cement and FA [23].

### 3.5. Porosity 

**Figure 7:**
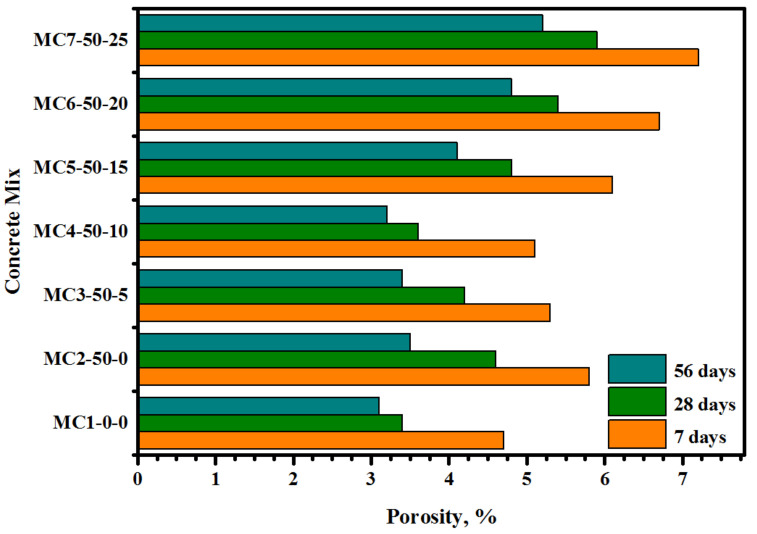
FA/EM contents-dependent porosity variation of the bio-concrete formulations.

**Figure 8:**
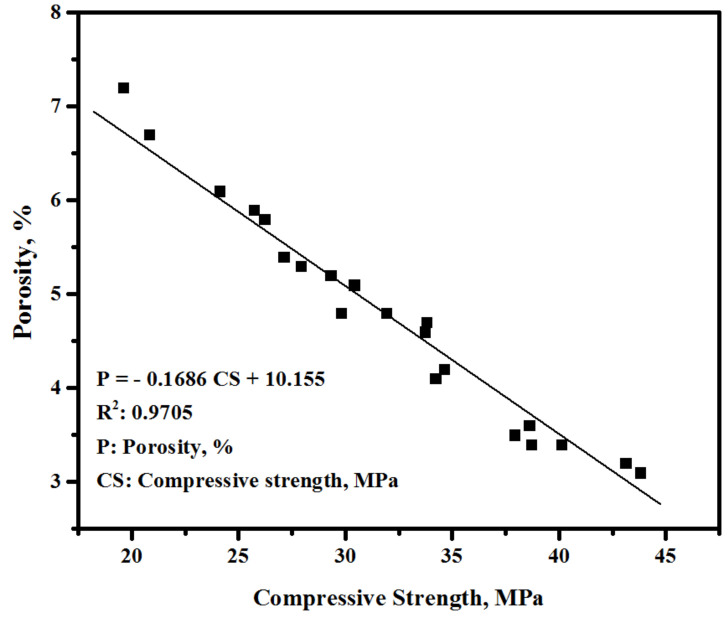
Porosity as a function of CS of bio-concretes at 7, 28 and 56 days of curing age.

Figure 7 displays the effect of the EM dosage on the porosity of the modified cement (cement-FA). For all concretes, the porosity percentage was decreased with the increase in the curing ages from 7 to 56 days. At 7 days (early age), the inclusion of 50% FA as the cement replacement was found to increase the porosity from 4.7 to 5.8%. However, with the increased curing ages of 28 and 56 days, the porosity of the bio-concretes dropped to 4.6% and 3.5%, respectively. The EM-activated bio-concretes showed much lower porosity. At 7 days, the replacement of tap water by 5 and 10% of EM could reduce the porosity of the bio-concrete from 5.8 to 5.1%, respectively. However, with the increase in the tap water replacement by EM from 15 to 25%, the porosity of the bio-concrete was slightly increased from 6.1% to 7.2%, respectively. Similar trends were observed after 28 and 56 days of curing age, wherein the increase in the EM dosage up to 10% could negatively affect the surface morphology of the bio-concrete, leading to an increase in porosity. At 28 days (late age), the bio-concrete with 10% EM as the replacement for the tap water achieved the optimum porosity, thus enhancing the surface morphology and reducing the total number of pores. The specimens prepared with 10% of EM displayed porosity readings of 3.6% and 3.2% compared to 4.6% and 3.5% at 28 and 56 days of age, respectively. The EM solution-incorporated mixes showed improved hydration reactions, indicating a decreased number of unreacted and partially-reacted particles with denser structures [52]. Regarding the enhanced surface morphology and the reduced density of the pores of the bio-concrete, an inverse relationship between the porosity (*P*) and CS was ascertained (Figure 8) with R^2^ = 0.97, indicating good confidence for the correlation obtained via:

*P* = −0.1686 CS + 10.155 (2)

### 3.6. Carbonation Depth

**Figure 9:**
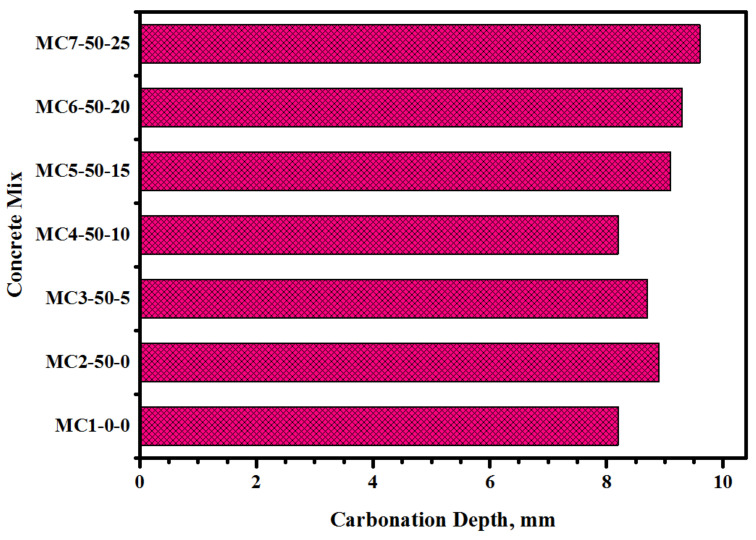
EM solution content-dependent CD of the bio-concrete formulations.

Figure 9 displays the EM solution content-dependent CD of the bio-concretes, indicating the significant effect of FA and EM solution on the CD values at 28 days of age. The replacement of the cement with 50% FA was found to adversely affect the surface morphology of the prepared bio-concrete, increasing the number of pores and, thus, the CD from 8.2 to 8.9 mm. However, the inclusion of EM solution into the cement-FA matrix positively affected the surface morphology of the prepared bio-concrete, reducing the CD. The addition of 5% and 10% of the EM solution as a replacement for the tap water was observed to reduce the CD from 8.7 to 8.2 mm, respectively. However, the further increase in the EM solution from 15% to 25% was shown to negatively influence the surface morphology of the prepared bio-concrete, increasing the CD from 9.1 to 9.6 mm, respectively. It was asserted that the inclusion of 10% EM solution in the concrete as a replacement for tap water significantly improved the durability performance of the bio-concrete by appreciably reducing the CD. It was also reported [45,52,53] that the number of void spaces can be lowered with the addition of an optimal quantity of pozzolanic materials as a cement substitution, which can be ascribed to the dense C-A-S-H gel formulation in the concrete matrix. In short, the reduction in the number of voids can positively reduce the porosity of the bio-concrete, thus lowering their CD.

### 3.7. Sulphuric Acid Resistance 

**Figure 10:**
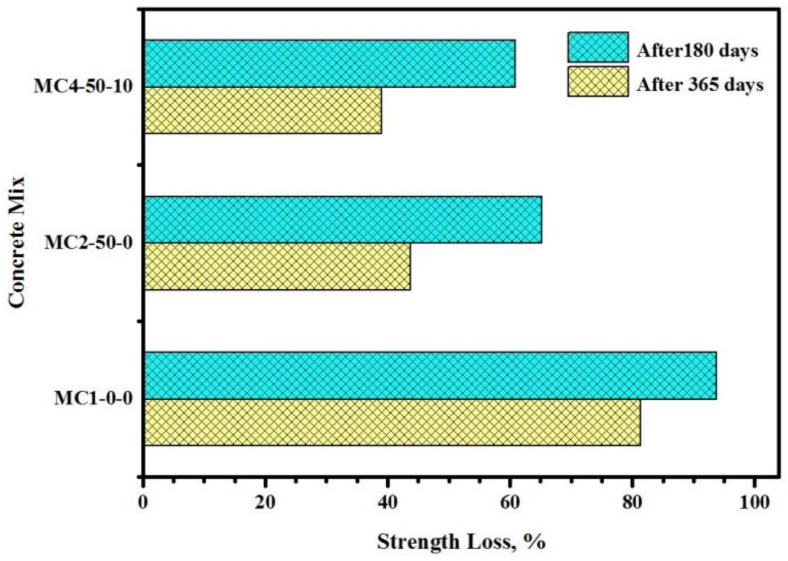
Influence of FA and EM on CS loss of the bio-concretes exposed to 10% of H_2_SO_4_ solution.

Figure 10 illustrates the influence of FA and EM on the CS loss of the bio-concretes exposed to 10% H2SO4 solution. It is important to mention that the benefits of using FA and EM in the bio-concrete to enhance the durability were evaluated in terms of their resistance against sulphuric acid attacks. The durability of the proposed bio-concretes was measured in terms of the residual CS, weight loss, internal crack formation using ultrasonic pulse velocity (UPV), virtual appearance, and microstructure properties. The loss in strength increased with the increase in the acid-exposure period (Figure 10). Bio-concrete exposed to the acid solution for a year showed lower performance compared to the one immersed for 180 days. The FA and EM-incorporated specimens displayed enhanced durability. The bio-concrete exposed to the acid solution for 180 days showed a strength decrease from 43.7 and 38.9% when prepared with 50% FA and 10% EM, respectively. Similar results were obtained after one year of acid solution exposure, wherein the strength decreased from 93.7% to 65.2% and 60.8% due to the corresponding addition of the FA and EM solution in the cement matrix. The enhanced durability of the modified bio-concrete can be attributed to the pozzolanic reactions of FA (50%) that led to the formation of Ca(OH)2 through the hydration reaction of the cement pastes producing excess hydrated products, such as C-S-H gels. Accordingly, both CS and the durability of the bio-concretes were enhanced because of the hardened specimen’s densification. Moreover, the constituents of FA, such as silicates and aluminates, were easily dissolved inside the concretes’ core due to its high alkalinity, inducing further pozzolanic reaction with the existing Ca in the cement matrix [2,4].

**Figure 11:**
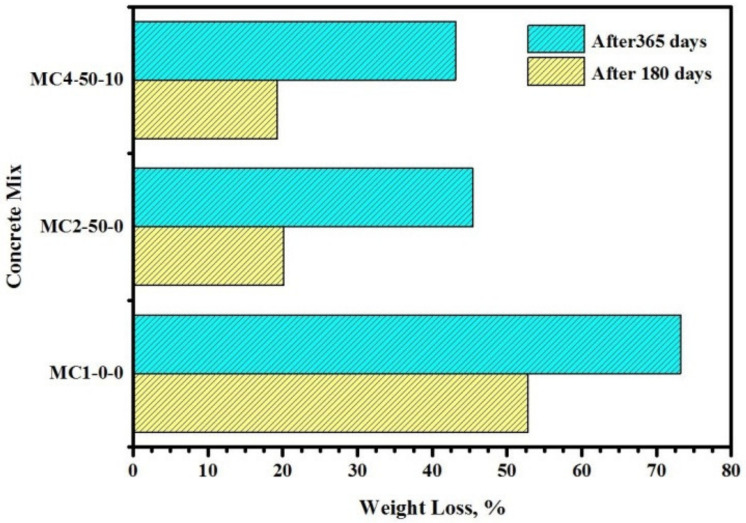
Influence of FA and EM on weight loss of the bio-concretes exposed to 10% of H_2_SO_4_ solution.

Figure 11 depicts the influence of FA and EM on the weight loss of the bio-concretes exposed to 10% H_2_SO_4_ solution. The weight loss of the bio-concretes was evaluated after 180- and 365-days of exposure to the acid solution. The results revealed that with the increase in the immersion period, the durability of proposed bio-concretes was reduced, which was mainly due to the formation of more internal cracks (Figure 11) and increased deterioration. However, the FA- and EM-included concrete showed reduced weight loss and improved durability. After 180 days, the FA- and EM-incorporated specimens showed a significant drop in the weight loss percentage from 20.1 and 19.3%, respectively. Similar trends were observed after 365 days of immersion in the acid solution, wherein the concretes designed using FA or FA and EM revealed better performance, with corresponding drops in weight loss from 45.4 and 43.1%, which were better than the control specimen. It was affirmed that the inclusion of FA and EM could lower the CaO content in the cement matrix, thus restricting the formulation of more Ca(OH)_2_ and gypsum. The gypsum formulation can generate extra internal stress and strain, leading to the development of more cracks and weight loss in the concrete [23].

**Figure 12:**
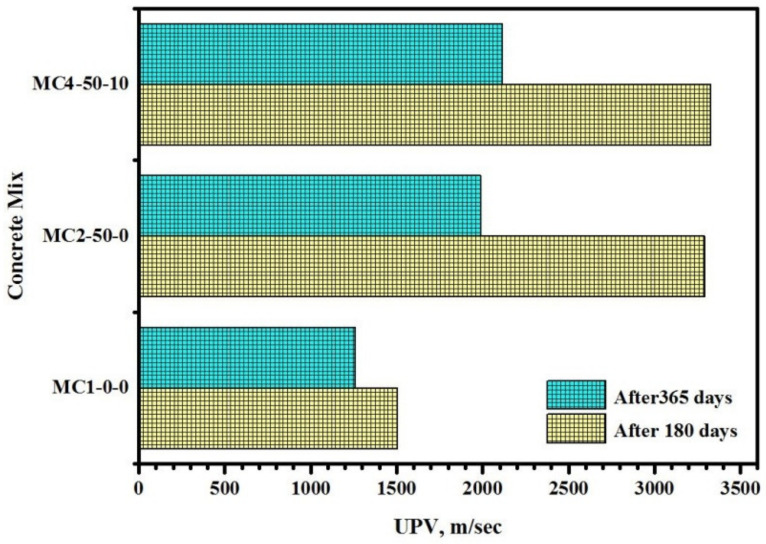
Influence of FA and EM on UPV readings of bio-concrete exposed to 10% of H_2_SO_4_ solution.

Figure 12 shows the UPV readings of the modified concrete containing FA and EM under 10% H_2_SO_4_ exposure for 180 days and one year. The interior and outside corrosion of all concretes were increased with increased exposure times. After 180 days of immersion, the concrete containing FA and EM showed fewer inner cracks, increasing the UPV reading from 1504.8 m/s to 3326.2 m/s. Similar results were observed after 365 days of immersion, wherein the specimen made from FA and EM showed an increase in the UPV reading from 1259.3 m/s to 2112.8 m/s. The lower UPV reading for the specimen exposed to acid for one year compared to the one exposed for 180 days was mainly due to the generation of more inner cracks that led to higher porosity, thus producing lower durability after prolonged exposure to the acid solution. In general, when FA and EM were included, the specimen showed fewer inner cracks and the formation of more stable gels in the acidic solution than in the pure OPC matrix. When the bio-concrete was subjected to the acid solution, the Ca(OH)_2_ reacted with the SO_4_^−2^ ion and produced CaSO_4_.2H_2_O. Consequently, the concrete networks were expanded and contained more inner cracks. This observation was consistent with the other findings [54,55,56,57], wherein it was demonstrated that more stable (C-(A)-S-H) gel forms due to an improvement in the aluminosilicates; these reductions, in turn, reduced the Ca(OH)_2_ and gypsum (Ca_2_SO_4_·2H_2_O) levels. The recorded loss in concrete mass and strength can be ascribed to acid molecule diffusion into the cement network matrix and subsequent gel damage. In addition, the soft and soluble gypsum strongly reacted with Ca(OH)_2_ and generated ettringite (3CaO·Al_2_O_3_·3CaSO_4_·32H_2_O). Nevertheless, the existence of FA and EM in the concrete matrix led to a reduction in unstable calcium, restricting the formation of gypsum and thus lowering the concrete spalling and deterioration [52,58,59].

**Figure 13:**
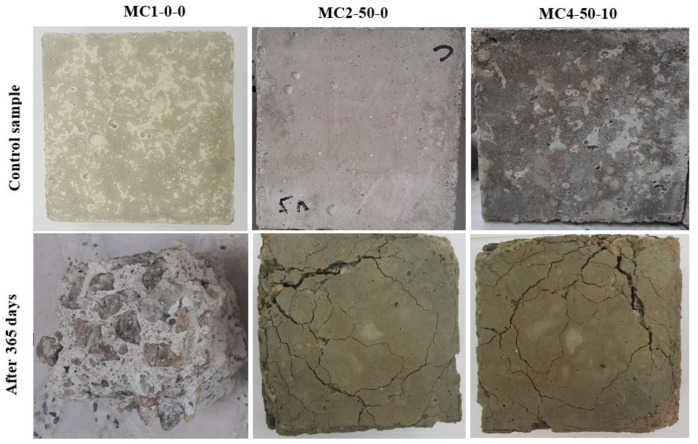
Visual appearance of modified concrete exposed H_2_SO_4_ after 365 days of immersion period.

Figure 13 shows the visual appearance of bio-concretes containing FA and EM solution after one year of 10% H_2_SO_4_ solution exposure. The resistance of the modified concrete to sulphuric acid attack was enhanced, as shown by the reduced external cracks, edges, and surface deterioration. The specimen devoid of FA and EM (control sample) showed the formation of larger cracks compared to the bio-concrete containing both FA (50%) and EM solution (10%). The bio-concrete corrosion was significantly decreased with the increased FA and EM levels of 50% and 10%, respectively. The inclusion of FA and EM into the concrete mixes could reduce the gypsum formulation as well as enabling the generation of more stable gels, improving the bio-concrete resistance and preventing deterioration.

**Figure 14:**
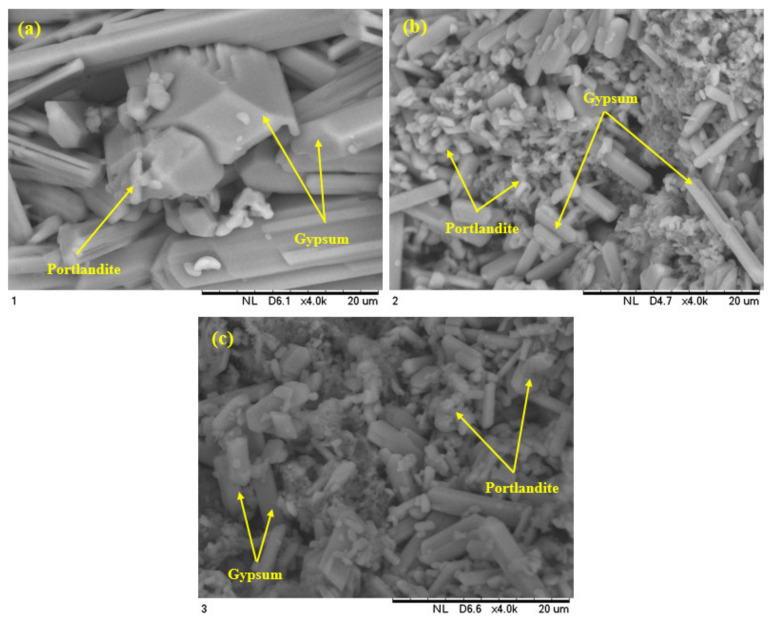
Effect of sulphuric acid solution on surface morphology of modified concrete specimen after 364 days of exposure (**a**) MC1-0-0 (**b**) MC2-50-0 and (**c**) MC4-50-10.

Figure 14 presents SEM images of the modified concretes under 10% H_2_SO_4_ solution exposure for one year. Specimens containing FA or FA and EM solution showed a decreased quantity of gypsum and presented with a lower deterioration compared to the cement-based concrete (control sample). The concrete specimens prepared with cement only (Figure 14a) displayed more gypsum and ettringite compared to the bio-concrete made with FA (50%) and EM solution (10%). The achieved homogeneous and dense structures of the EM solution-activated concrete clearly verified the existence of unreacted and partially reacted silica (Figure 14b,c). The SEM results (Figure 14a) revealed the formation of many rod-shaped CaSO_4_·H_2_O crystals (gypsum) [60,61]. Generally, the bio-concrete made from FA and EM solution showed lower gypsum and ettringite levels than the pure OPC-based concrete. The inclusion of FA and EM solution into the cement matrix could appreciably improve the bio-concrete resistance against the acid attack. The lower Ca(OH)_2_ level in the bio-concrete was responsible for the improved acid attack resistance and enhanced durability.

### 3.8. Reduction of Carbon Dioxide Emission 

**Figure 15:**
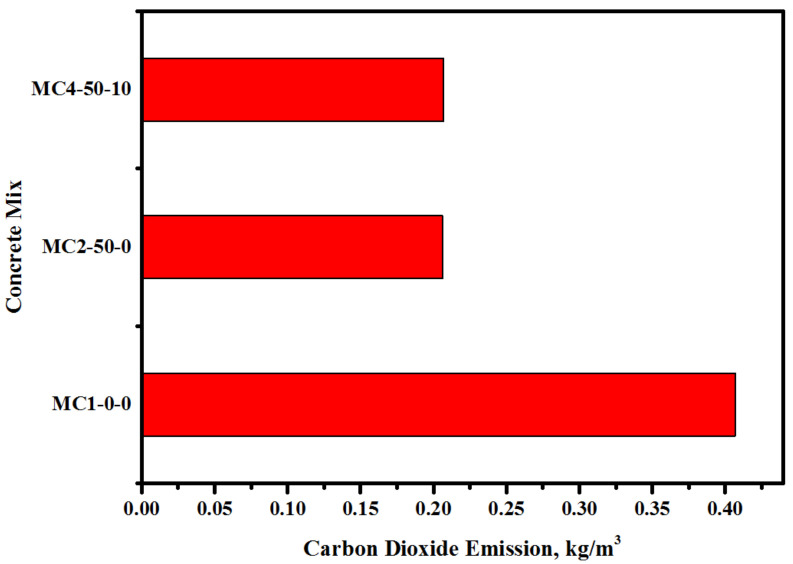
Effect of FA and EM content on CO_2_ emission of the modified concretes.

The environmental benefits of the FA and EM solution-incorporated bio-concrete were evaluated in terms of the predicted lowering of carbon dioxide emissions. Figure 15 displays the effect of FA and EM content on the CO_2_ emissions of the modified concrete. Table 6 shows the transportation distance and fuel consumed by every material that was used in the analysis. The inclusion of 50% FA into the cement matrix was found to significantly reduce the total CO_2_ emissions, achieving substantial environmental benefits. The inclusion of FA and EM solution into the cement matrix was found to reduce the CO_2_ emissions from 0.4068 kg/m^3^ to 0.2067 kg/m^3^. The proposed concrete achieved much lower carbon emissions (above 49.3%) compared to the pure OPC-based control specimen. Conversely, the inclusion of 50% FA as a cement replacement in the high-performance bio-concrete was shown to reduce the demand for natural resources as well landfill problems related to the disposal of hazardous waste materials.

## 4. Conclusions

In this work, the possibility of producing sustainable bio-concrete using FA and EM with improved strength and durability performance was explored. From the obtained results, the following conclusions were derived:

i. The replacement of tap water with the EM solution led to a reduced pH and surface tension. However, the viscosity of EM solution was increased with the increase in the EM level. The fresh and hardened properties of bio-concrete were significantly influenced by the inclusion of the EM solution.
ii. The inclusion of 50% FA as an OPC replacement could directly affect the early and late strength development of the proposed bio-concrete.
iii. The inclusion of 5% and 10% EM in the cement-FA matrix improved the CS at early and late ages.
iv. The highest CS was achieved for the bio-concrete prepared with 10% EM. The EM solution improved the hydration process and led to the formulation of denser gels, thus yielding a better performance compared to other dosages of EMs.
v. The SEM results showed that the replacement of tap water with 10% EM solution could improve the surface morphology of the bio-concrete and reduce the number of pores. This, in turn, increased the strength and reduced the porosity of the bio-concrete. An inverse relationship was observed between strength and porosity of modified concretes.
vi. The reduction in the porosity and total number of pores in the modified concrete prepared with 10% EM could contribute to the improvement of their durability by reducing the carbonation depth.
vii. The inclusion of FA and EM in the cement matrix led to an increase in the concrete resistance during sulphuric acid attack in terms of the reduced strength loss, weight loss, internal cracks, and deterioration of both the surface and edges.
viii. Overall, the FA and EM solution-incorporated concrete showed a great potential for construction applications. It is asserted that such bio-concretes may offer a possible solution to reduce the reliance on standard OPC-based concretes that contribute appreciably to pollution, promoting the development of materials of greater sustainability and thus minimizing the negative effect on the environment.

## Figures and Tables

**Table 1:** Chemical compositions of OPC and FA obtained from XRF analyses.

Raw Materials	Elements (Weight%)								
	SiO_2_	Al_2_O_3_	Fe_2_O_3_	CaO	MgO	K_2_O	Na_2_O	SO_3_	LOT
OPC	20.4	5.2	4.2	62.4	1.6	0.01	0.2	2.1	2.4
FA	57.2	28.8	3.7	5.2	1.5	0.9	0.1	0.1	0.1

**Table 2:** Physical traits of FA and OPC.

Properties	FA	OPC	Permissible Limits	Relevant Standard
Specific gravity	2.20	3.15	3.10–3.25	ASTM C33
Color	Grey	Dark Grey	-	-
%Passing through 45 μm wet sieve	100	90	≥ 34	ASTM C430

**Table 3:** Modified concrete mix design containing different amount of EM.

Mix	Binder, kg/m^3^			Solution, kg/m^3^		Aggregates, kg/m^3^	
	OPC	FA	CaO:SiO_2_	Water	EM	Sand	Gravel
MC1-0-0	450	0	3.06	250	0	875	815
MC2-50-0	225	225	0.92	250	0	875	815
MC3-50-5	225	225	0.92	237.5	12.5	875	815
MC4-50-10	225	225	0.92	225	25	875	815
MC5-50-15	225	225	0.92	212.5	37.5	875	815
MC6-50-20	225	225	0.92	200	50	875	815
MC7-50-25	225	225	0.92	225	62.5	875	815

**Table 4:** Prepared EM solution properties (pH, viscosity, and surface tension).

Properties	EM Solution	Water Replacement by EM Solution (%)					
		0	5	10	15	20	25
pH	3.45	6.7	6.4	6.3	5.7	5.2	4.0
Viscosity, mPas	1.44	0.95	1.0	1.05	1.1	1.2	1.25
Surface tension, mN/m	66	66	58.3	54.5	51.7	44.8	39.9

**Table 5:** The XRD peak values for modified concrete with FA and EM, weight %.

Index	MC1-0-0	MC2-50-0	MC4-50-10
Ca(OH)_2_	13.9	8.4	18.4
SiO_2_	68.6	76.3	67.5
CaCO_3_	8.8	6.1	9.2
Ca_6_Al_2_(SO_4_)_3_(OH)_12_·26H_2_O	7.1	1.3	2.1

**Table 6:** OPC, FA and EM materials carbon dioxide emission different components of the life cycle.

Transportation Cost Parameters			
Speed, km/h	Diesel consumption, liter/km	Truck volume, m^3^	CO_2_ emission for 1 L diesel, ton
80	0.09	12	0.0027
Materials CO_2_ emission, energy consumption and cost.			
Materials	Manufacturing (CO_2_, t/t)	Transportation (CO_2_, t/t)	Total (CO_2_, t/t)
OPC	0.9023	0.0017	0.904
FA	0	0.012	0.012
EM	0	0	0

## Data Availability

Not applicable.

## References

[1] Talaiekhozan A., Majid M.Z.A. (2014). A review of self-healing concrete research development. J. Environ. Treat. Tech..

[2] Huseien G.F., Joudah Z.H., Memon R.P., Sam A.R.M. (2021). Compressive strength and microstructure properties of modified concrete incorporated effective microorganism and fly ash. Mater. Today Proc..

[3] Huseien G.F., Sam A.R.M., Algaifi H.A., Alyousef R. (2021). Development of a sustainable concrete incorporated with effective microorganism and fly Ash: Characteristics and modeling studies. Constr. Build. Mater..

[4] Wu M., Johannesson B., Geiker M. (2012). A review: Self-healing in cementitious materials and engineered cementitious composite as a self-healing material. Constr. Build. Mater..

[5] Jonkers H.M. (2007). Self healing concrete: A biological approach. Self Healing Materials.

[6] Jonkers H.M., Thijssen A., Muyzer G., Copuroglu O., Schlangen E. (2010). Application of bacteria as self-healing agent for the development of sustainable concrete. Ecol. Eng..

[7] Van Tittelboom K., De Belie N., De Muynck W., Verstraete W. (2010). Use of bacteria to repair cracks in concrete. Cem. Concr. Res..

[8] Nathaniel O., Sam A.R.M., Lim N.H.A.S., Adebisi O., Abdulkareem M. (2020). Biogenic approach for concrete durability and sustainability using effective microorganisms: A review. Constr. Build. Mater..

[9] Hamzah N., Saman H.M., Baghban M.H., Sam A.R.M., Faridmehr I., Sidek M.N.M., Benjeddou O., Huseien G.F. (2022). A Review on the Use of Self-Curing Agents and Its Mechanism in High-Performance Cementitious Materials. Buildings.

[10] Hemsley R.A., Griffiths P.C. (2000). Architecture in the microcosm: Biocolloids, self-assembly and pattern formation. Philos. Trans. R. Soc. Lond. Ser. A Math. Phys. Eng. Sci..

[11] Huseien G.F., Shah K.W., Sam A.R.M. (2019). Sustainability of nanomaterials based self-healing concrete: An all-inclusive insight. J. Build. Eng..

[12] Isa M.N., Garba M.M., Kawu A.L. (2016). Influence of locally made effective microorganisms on the compressive strength of concrete. J. Multidiscip. Eng. Sci.Technol..

[13] Rong H., Qian C.-X., Li L.-Z. (2012). Study on microstructure and properties of sandstone cemented by microbe cement. Constr. Build. Mater..

[14] Ghosh S., Biswas M., Chattopadhyay B., Mandal S. (2009). Microbial activity on the microstructure of bacteria modified mortar. Cem. Concr. Compos..

[15] Sato N., Higa T., Sugita S., Shuya. M. Some properties of concrete mixed with effective microorganisms and the on-site investigation of the completed structures. Proceedings of the 28th International Conference, Our World in Concrete and Structures.

[16] Andrew T.C.S., Syahrizal I.I., Jamaluddin M.Y. Effective microorganisms for concrete (EMC) admixture–its effects to the mechanical properties of concrete. Proceedings of the Awam International Conference on Civil Engineering (AICCE’12) Geohazard Information Zonation (GIZ’12).

[17] Rizwan S.A., Khan H., Bier T.A., Adnan F. (2017). Use of Effective Micro-organisms (EM) technology and self-compacting concrete (SCC) technology improved the response of cementitious systems. Constr. Build. Mater..

[18] Yu Z., Zhang J., Zhao X., Zhao X., Duan J., Song X. (2014). Effects of microorganism on corrosion performance of zinc in natural seawater. Int. J. Electrochem. Sci..

[19] Zhai X., Ren Y., Wang N., Guan F., Agievich M., Duan J., Hou B. (2019). Microbial Corrosion Resistance and Antibacterial Property of Electrodeposited Zn–Ni–Chitosan Coatings. Molecules.

[20] Nelson V.V., Maria O.T., Mamiè S.V., Maritza P.C. (2017). Microbiologically influenced corrosion in aluminium alloys 7075 and 2024, in Aluminium Alloys-Recent Trends in Processing, Characterization, Mechanical Behavior and Applications. IntechOpen.

[21] Huseien G.F., Nehdi M.L., Faridmehr I., Ghoshal S.K., Hamzah H.K., Benjeddou O., Alrshoudi F. (2022). Smart Bio-Agents-Activated Sustainable Self-Healing Cementitious Materials: An All-Inclusive Overview on Progress, Benefits and Challenges. Sustainability.

[22] Memon R.P., Mohd A.R.B., Awang A.Z., Huseien G.F., Memon U. (2018). A review: Mechanism, materials and properties of self-curing concrete. ARPN J. Eng. Appl.Sci..

[23] Huseien G.F., Joudah Z.H., Khalid N.H.A., Sam A.R.M., Tahir M.M., Lim N.H.A.S., Alyousef R., Mirza J. (2021). Durability performance of modified concrete incorporating fly ash and effective microorganism. Constr. Build. Mater..

[24] Iriti M., Scarafoni A., Pierce S., Castorina G., Vitalini S. (2019). Soil application of effective microorganisms (EM) Maintains leaf photosynthetic efficiency, increases seed yield and quality traits of bean (*Phaseolus vulgaris* L.) plants grown on different substrates. Int. J. Mol.Sci..

[25] Cabrera M., Galvín A., Agrela F. (2019). Leaching issues in recycled aggregate concrete. New Trends in Eco-Efficient and Recycled Concrete.

[26] Wang H., Li Q. (2007). Prediction of elastic modulus and Poisson’s ratio for unsaturated concrete. Int. J. Solids Struct..

[27] Rashad A.M., Ezzat M. (2019). A Preliminary study on the use of magnetic, Zamzam, and sea water as mixing water for alkali-activated slag pastes. Constr. Build. Mater..

[28] Zhang J., Dong B., Hong S., Teng X., Li G., Li W., Tang L., Xing F. (2019). Investigating the influence of fly ash on the hydration behavior of cement using an electrochemical method. Constr. Build. Mater..

[29] Liu J., Qiu Q., Xing F., Pan D. (2014). Permeation properties and pore structure of surface layer of fly ash concrete. Materials.

[30] Yu Z., Ye G. (2013). The pore structure of cement paste blended with fly ash. Constr. Build. Mater..

[31] Shaikh F.U., Supit S.W. (2015). Compressive strength and durability properties of high volume fly ash (HVFA) concretes containing ultrafine fly ash (UFFA). Constr. Build. Mater..

[32] Wang H., Li H., Liang X., Zhou H., Xie N., Dai Z. (2019). Investigation on the mechanical properties and environmental impacts of pervious concrete containing fly ash based on the cement-aggregate ratio. Constr. Build. Mater..

[33] Cho Y.K., Jung S.H., Choi Y.C. (2019). Effects of chemical composition of fly ash on compressive strength of fly ash cement mortar. Constr. Build. Mater..

[34] Sathyan D., Anand K.B. (2019). Influence of superplasticizer family on the durability characteristics of fly ash incorporated cement concrete. Constr. Build. Mater..

[35] Gulbandilar E., Kocak Y. (2013). Prediction of the effects of fly ash and silica fume on the setting time of Portland cement with fuzzy logic. Neural Comput. Appl..

[36] Mhaya A.M., Baghban M.H., Faridmehr I., Huseien G.F., Abidin A.R.Z., Ismail M. (2021). Performance evaluation of modified rubberized concrete exposed to aggressive environments. Materials.

[37] Zhang R., Shi N., Huang D. (2013). Influence of initial curing temperature on the long-term strength of concrete. Mag. Concr. Res..

[38] Lothenbach B., Scrivener K., Hooton R. (2011). Supplementary cementitious materials. Cem. Concr. Res..

[39] Al-Fasih M.Y.M., Huseien F.G., bin Ibrahim S.I., Sam M.A.R., Algaifi A.H., Alyousef R. (2021). Synthesis of rubberized alkali-activated concrete: Experimental and numerical evaluation. Constr. Build. Mater..

[40] Nedunuri S.S.S.A., Sertse S.G., Muhammad S. (2020). Microstructural study of Portland cement partially replaced with fly ash, ground granulated blast furnace slag and silica fume as determined by pozzolanic activity. Constr. Build. Mater..

[41] Zuo W., Feng P., Zhong P., Tian Q., Gao N., Wang Y., Yu C., Miao C. (2017). Effects of novel polymer-type shrinkage-reducing admixture on early age autogenous deformation of cement pastes. Cem. Concr. Res..

[42] Huseien G., Ismail M., Tahir M., Mirza J., Hussein A., Khalid N., Sarbini N. (2018). Performance of sustainable alkali activated mortars containing solid waste ceramic powder. Chem. Eng. Trans..

[43] Sun Y., Yu R., Shui Z., Wang X., Qian D., Rao B., Huang J., He Y. (2019). Understanding the porous aggregates carrier effect on reducing autogenous shrinkage of Ultra-High Performance Concrete (UHPC) based on response surface method. Constr. Build. Mater..

[44] Wehbe Y., Ghahremaninezhad A. (2017). Combined effect of shrinkage reducing admixtures (SRA) and superabsorbent polymers (SAP) on the autogenous shrinkage, hydration and properties of cementitious materials. Constr. Build. Mater..

[45] Zhan P.-M., He Z.-H. (2019). Application of shrinkage reducing admixture in concrete: A review. Constr. Build. Mater..

[46] Ali B., Qureshi L.A., Baig H.S., Malik S., Din M., Aslam H.M.U. (2020). Effect of molasses and water–cement ratio on properties of recycled aggregate concrete. Arab. J. Sci. Eng..

[47] Qin R., Hao H., Rousakis T., Lau D. (2019). Effect of shrinkage reducing admixture on new-to-old concrete interface. Compos. Part B: Eng..

[48] Dang Y., Qian J., Qu Y., Zhang L., Wang Z., Qiao D., Jia X. (2013). Curing cement concrete by using shrinkage reducing admixture and curing compound. Constr. Build. Mater..

[49] Ngene B.U., Olofinnade O.M., Agomo C.E. (2019). Effect of magnetized water on the mechanical properties of concrete containing recycled waste glass aggregate. International Journal of Engineering Research in Africa.

[50] Souza M.T., Onghero L., Correa B.N., Selhorst M.A., Dias A.M., Repette W.L., Pereira F.R., de Oliveira A.P.N. (2020). Novel low-cost shrinkage-compensating admixture for ordinary Portland cement. Constr. Build. Mater..

[51] Akar C., Canbaz M. (2016). Effect of molasses as an admixture on concrete durability. J. Clean. Prod..

[52] Mohammadhosseini H., Lim N.H.A.S., Tahir M.M., Alyousef R., Alabduljabbar H., Samadi M. (2019). Enhanced performance of green mortar comprising high volume of ceramic waste in aggressive environments. Constr. Build. Mater..

[53] Liew K., Sojobi A., Zhang L. (2017). Green concrete: Prospects and challenges. Constr. Build. Mater..

[54] Ahmed M.A.B., Hussin M.W., Muthusamy K., Ismail M.A. (2010). Performance of high strength POFA concrete in acidic environment. Concr. Res. Lett..

[55] Ariffin M., Bhutta M., Hussin M., Tahir M.M., Aziah N. (2013). Sulfuric acid resistance of blended ash geopolymer concrete. Constr. Build. Mater..

[56] Bamaga S., Ismail M.A., Majid Z.A., Ismail M., Hussin M.W. (2013). Evaluation of sulfate resistance of mortar containing palm oil fuel ash from different sources. Arab. J. Sci. Eng..

[57] Noruzman A., Ismail M., Bhutta M.A.R., Yusuf T.O., Shehu I.A., Hassan I.O. (2013). Strength and durability characteristics of polymer modified concrete incorporating Vinyl acetate effluent. Advanced Materials Research.

[58] Huseien G.F., Shah K.W. (2020). Durability and life cycle evaluation of self-compacting concrete containing fly ash as GBFS replacement with alkali activation. Constr. Build. Mater..

[59] Mohammadhosseini H., Yatim J.M., Sam A.R.M., Awal A.A. (2017). Durability performance of green concrete composites containing waste carpet fibers and palm oil fuel ash. J. Clean. Prod..

[60] Vafaei M., Allahverdi A., Dong P., Bassim N. (2018). Acid attack on geopolymer cement mortar based on waste-glass powder and calcium aluminate cement at mild concentration. Constr. Build. Mater..

[61] Slaty F., Khoury H., Rahier H., Wastiels J. (2015). Durability of alkali activated cement produced from kaolinitic clay. Appl. Clay Sci..

